# Tourists’ Fascination with Urban Food Markets: The Successful Case of Time Out Market Lisbon

**DOI:** 10.3390/foods12091795

**Published:** 2023-04-26

**Authors:** Arlindo Madeira, Rosa Rodrigues, Teresa Palrão, Alexandra Sofia Mendes

**Affiliations:** 1 Faculty of Social Sciences and Technology, Universidade Europeia, 1749-016 Lisboa, Portugal; arlindo.madeira@universidadeeuropeia.pt; 2 ESCAD—Escola Superior de Ciências da Administração, IPLUSO, 1200-427 Lisboa, Portugal; 3 Business & Economics School, Instituto Superior de Gestão, 1700-284 Lisboa, Portugal; rosa.rodrigues@isg.pt; 4 CEFAGE—Center for Advanced Studies in Management and Economics, 7000-809 Évora, Portugal; 5 ISCE—Instituto Superior de Lisboa e Vale do Tejo, 2620-379 Ramada, Portugal; teresa.palrao@isce.pt; 6 CiTUR—Centre for Tourism Research, Development and Innovation, 2520-614 Peniche, Portugal; 7 School of Tourism and Maritime Technology, Polytechnic of Leiria, 2520-614 Peniche, Portugal

**Keywords:** food neophilia, Time Out Market Lisbon, cultural attraction, visitor’s satisfaction, intention to revisit

## Abstract

This research was designed to analyze tourists’ perception of food markets in an urban context, specifically in the case of Time Out Market Lisbon. The sample included participants who visited and experienced the market food court. The data were collected using a questionnaire assessing the respondents’ perceptions of the location, food quality, food neophilia, market engagement, and the cultural attractiveness of the locale. The purpose was also to analyze how these factors contribute to visitor satisfaction and their intention to revisit the local area. The findings showed that food neophilia was the characteristic of Time Out Market Lisbon that had the highest impact on visitors’ satisfaction which, accordingly, positively influenced their intention to revisit the market. Visitors’ perception of the place as a cultural attraction was also found to moderate the relationship between visitors’ satisfaction and intention to revisit Time Out Market Lisbon. The findings provide an important contribution to the scientific community regarding the important role of modern food markets in urban city regeneration and more specifically as a tourist attraction. This research has significant management implications regarding the emotions of tourists’ food experience and can be used for stakeholders engaged in the tourism development process.

## 1. Introduction

Gastronomy plays an important role in visitor satisfaction, enhancing the image of the destination and improving its economy, especially in those where the gastronomic offer is richer [1]. Thus, tourism decision-makers, being conscious of this reality, have been promoting gastronomy as a tourism product in destinations [2]. Tourists, who have gastronomic experiences as their main motivation for travel still constitute a niche market [3]. The gastronomic tourists are motivated by the desire to understand the residents’ way of life through the local cuisine [4]. However, gastronomy as a tourist attraction accommodates all kinds of travel motivations because all travelers need to eat [5]. Therefore, all travelers end up being food tourists, regardless of their reason for visiting a destination [6].

Regarding gastronomic tourism in an urban context, this reality is even more present, since cities offer multi-product tourism, in contrast to rural destinations, which, in general, have less incentives to attract visitors [7,8]. Accordingly, the concept of gastronomic cities has been gaining momentum worldwide, through successful projects and case studies where gastronomy is at the center of the urban strategy for tourism and employment development [9]. In practice, besides the multiple types and concepts of restaurant that cities offer, urban tourism has other particularities such as the large-scale adhesion to gastronomic festivals, which promote regional and international cuisines and, more recently, food and gastronomic markets [10].

Food markets have been key forces in the structural and economic development of western European cities even since the classical Mediterranean civilizations [11]. Contemporary urban food markets are a recent manifestation of the ancient Greek agoras or Roman forums [12,13]. Since the end of the last century, across Europe, these markets, once vibrant and essential for supplying cities and their inhabitants, have come to be seen as an outdated and obsolete form of retail, having lost their former relevance [14]. However, in the last decade, these European city landmarks have started to be reborn, thanks to a new approach. Food markets, once in decline given the dominance of hypermarkets and shopping centers, gained new life with the inclusion of catering establishments, which cohabitate with the stalls where local products are sold [8].

Gastronomic tourism in urban spaces adds not only the food and drink attraction, but also historical and social attractions. These urban markets, with a historical role rooted in people’s lives, are a tourist attraction for the visitor, where they can experience both indigenous products and the authenticity of the place and its people [15]. The combination of factors that mixes gastronomy, urbanism, and tourism must be achieved through a sustainable balance that benefits the inhabitants, the visitors, and, naturally, the promoters [16].

The present study focuses on the Time Out Market Lisbon, considered an international success story, which boosted the opening of other brand spaces, based on the same business model. The gastronomic market cohabitates with the traditional market, where sellers trade daily food products such as vegetables, fruits, fish, and meat. The gastronomic and food market presents a mix of gastronomic venues, based on Portuguese cuisine, ranging from traditional food to fine dining cuisine, as well as dance and shows, which mostly attract tourists. The objectives of the study are: (i) to understand the fascination that this urban trade space exerts over visitors to the city and (ii) to understand which factors have the greatest positive impact on the experience that the market offers.

In Dimitrovski and Crespí-Vallbona’s model [17] on which this study was based, food neophilia was tested as a moderator variable for satisfaction and cultural experience, i.e., the influence that food neophilia has on the relationship between cultural experience and satisfaction, and between market involvement and satisfaction. Thus, this study responds to this literature gap by assessing the direct influence of food neophilia on satisfaction, which had not been tested before. The findings showed that food neophilia was the variable had the most positive influence on visitor satisfaction. Furthermore, the present research included the market location as a factor that influenced the overall visit.

## 2. Theoretical Background

### 2.1. Urban Food Markets and Tourism

Markets have been a critical element in the growth of cities and human settlements throughout its history, due to its capacity to profoundly change the vitality and viability of urban centers [14,18]. Besides their function of warehousing and selling food products, markets have always performed a socializing role as public spaces, where traders, residents, and visitors interact simultaneously [19]. This social capacity of food markets enhances tourist visits and contributes to the revitalization of places, especially those that have been transformed into gastronomy markets [14]. That is, traditional markets, now revitalized and reconverted into gastronomy markets, have facilitated the development of the process of urban regeneration, as new tourist attractions [15]. Furthermore, visitors choose to discover these spaces, not only to have unique gastronomic experiences and social interactions, but also to discover the architecture, history, and culture of each market, and to feel the local atmosphere [17].

### 2.2. Food Quality in Urban Food Markets

Contemporary consumers are increasingly looking for native ingredients and quality food products [20]. This trend also extends to tourists who have a notorious interest in local food as it represents the typicality and identity of a particular place [16]. That is, the quality and freshness of food products are attractive for both locals and tourists [21]. Gastronomic tourism experiences happen not only in restaurants, but also through visits to food markets [22]. Therefore, to be recognized by tourists, food markets must be authentic and genuine [16]. In relation to gastronomic markets where both the sale of food products and restaurant spaces coexist, the experience is maximized by the local gastronomic offer, especially when presented with high quality concepts [15]. Thus, quality gastronomy is one of the most important assets for tourist destinations, because it promotes participative experiences that bring people closer to the local culture [10].

### 2.3. Urban Food Market’s Location

The location of markets in city centers has always been a strategic factor for supplying the population, as it allows for greater efficiency in the movement of inhabitants [23]. Furthermore, due to its location, food markets were, since the beginning, one of the meeting and socializing points in cities [24]. The rebirth of historic city centers, in recent decades, enabled the rediscovery and the regeneration of European urban marketplaces, both by locals and tourists [25]. Tourists like to visit the historic centers for the unique architectural layout of their streets and buildings, but also because it allows them to experience and mingle with the locals [17]. Food consumption does have a highly noticeable and crucial role in the life of a city and in the design of welcoming public spaces, as food acts as a vehicle that allows for the sustainment and socialization of urban spaces [26]. The type of visitor who likes to explore historic centers values culture, culinary art, and social interactions and escapes the crowds to seek out the more touristy neighborhoods, where markets are usually located [27]. Hence, typical markets in city centers combine genuine experiences made of interpersonal interactions with the local population and culture, food, and local gastronomy [25].

### 2.4. Food Market Involvement

The food markets’ involvement is achieved through the stalls, food, people’s interactions, and the mixture of aromas and colors that each space presents us [17]. These gastronomic experiences in urban places have their essence in the production of social relationships resulting from the interaction of customers (tourists and residents) with vendors, creating points of identification with the place [28]. That is, the cultural aspects related to each food market define the atmosphere and environment as identifying marks of the place [17]. Hence, tourists, when exploring other cuisines, become culturally involved with the place and its people, through the gastro-festive environment that surrounds each market [29]. The involvement of tourists with the market atmosphere also extends to the places where markets are built, usually historic centers, which constitute an added value to the experience [30]. Urban food markets and their surroundings are therefore places where tourists create affective connections with the surrounding environment, which contribute to the overall satisfaction of the trip [17].

### 2.5. Revisit Intention and Gastronomy

The importance of gastronomy in the intention to revisit has been widely studied in the context of gastronomic tourism [31,32,33]. It appears that experiences with local cuisines add value to the overall traveling experience, in such a way that many tourists consider returning to a specific destination to taste its gastronomy [31]. These previous experiences relate the place of consumption with the cuisine, art, and local culture, contributing to a favorable image of the destination [34]. In this sense, tourists who decide to revisit the destination have a more positive attitude towards the local food because of their prior experience and familiarity with local cuisines [35]. The influence of gastronomy in revisiting the destination is related to the security and confidence acquired in previous positive experiences [36]. Due to its natural aptitude, which combines local cuisine with art and culture, food markets are privileged places for the promotion of unique gastronomic experiences [24].

### 2.6. Food Neophilia and Tourism

From the tourist’s perspective, experiencing the unique food of a destination is both a necessity and a pleasure [37]. The search for new gastronomic experiences is one of the most important motivations for tourists [38]. Food neophilia is based on the trend to try new foods, commonly known as novelty seeking [17]. Tourists as neophiliacs are more likely to experience positive emotions through unfamiliar foods and flavors that they recognize as typical of the destination [39]. Food neophiliacs view the experience of seeking out new flavors and ingredient combinations as a positive adventure, and as a natural part of the trip [40] and will try any kind of food since their choices are mainly generated by curiosity [41]. Hence, experiencing local cuisine novelty ends up making a decisive contribution to the positive experience at the destination [5]. Considering the positive relationship between food neophilia and tourists’ satisfaction, genuine food markets play a key role as showcase places for indigenous gastronomy [42]. Hence, the first hypothesis was elaborated:

**H1:**  *Food neophilia is the characteristic of Time Out Market Lisbon that most positively influences visitor satisfaction.*

### 2.7. Food Market Visitors’ Satisfaction

Visitor’s satisfaction is seen as a determining factor for the success of food markets, by defining the operational results of the business as a common space that houses several individual businesses [30]. Positive gastronomic experiences lead to satisfaction with destinations and their images [5]. Furthermore, satisfaction with gastronomic experiences leads to positive word of mouth and shapes future travel behavior [24,32]. Among the determining factors for the visitor´s satisfaction the following stand out: (i) the quality of food products [21]; (ii) the architectural and historical framework of the market and its surroundings [17]; (iii) the authenticity of the market, as a space that reflects the cultural dimension of the destination; and (iv) the pleasure of visitors in tasting different products [39]. Thus, food markets with high levels of satisfaction regarding the various dimensions of the visit, induce memorable experiences [24]. Accordingly, we propose that visitors’ satisfaction is a positive predictor of the intention to revisit Time Out Market Lisbon (second hypothesis).

**H2:**  *The intention to revisit Time Out Market Lisbon is positively influenced by visitors’ satisfaction.*

### 2.8. Food Market as Cultural Attraction

Local food markets are a key cultural attraction for tourists, due to the uniqueness and authenticity of these spaces, which express the destination’s way of life [43]. The uniqueness of each food market is emphasized by cultural differences and local history, including the indigenous food varieties and food preferences of each people [20]. Hence, tourists see food markets as a cultural showcase of the destination they visit and want to discover [44]. As a result, historic urban food markets become one of the cultural assets of cities, especially those that have become gastronomic markets, where people can taste local cuisine while relaxing and absorbing local culture [24]. In addition, food markets as cultural attractions are becoming places to promote types of minority tourism, as they bring together a set of attractions that go beyond gastronomy [29]. Thus, markets as new tourist spaces can improve the cultural experiences of travelers [17]. Thus, we propose our third hypothesis:

**H3:**  *The place as a cultural attraction moderates the relationship between visitors’ satisfaction and intention to revisit Time Out Market Lisbon.*

## 3. Research Background

**Figure 1:**
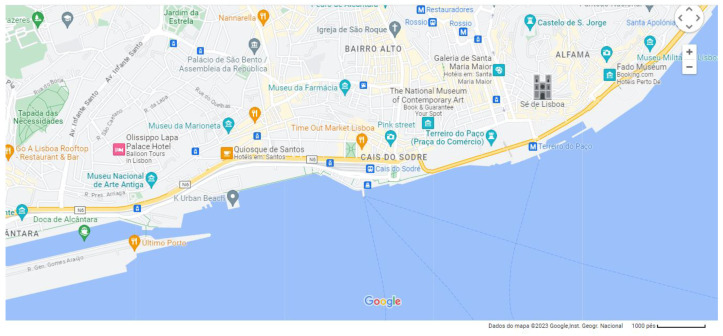
Time Out Market Lisbon location.

The Ribeira market is, since its inauguration in 1892, the main market in Lisbon (Figure 1). Despite having been subject to several renovations throughout its existence, the Ribeira market entered the 21st century without the glow of previous times. Like many other European urban markets, the Ribeira market was in decline due to by changing consumption patterns, namely with the spread of hypermarkets during the 1980s and 1990s [14]. The renaissance came in 2014 when it started to be managed by Time Out Lisbon magazine, which divided the market into traditional catering stalls, with a food court of diverse but exclusive culinary concepts, based on Portuguese cuisine. This gastronomic market concept, created by the Time Out Portugal team, would later be replicated in other cities, namely Miami, New York, Boston, Montreal, Chicago, and Dubai, with more openings in Porto in 2023 followed by Abu Dhabi already being scheduled for 2024 and Osaka for 2025 [45].

**Figure 2:**
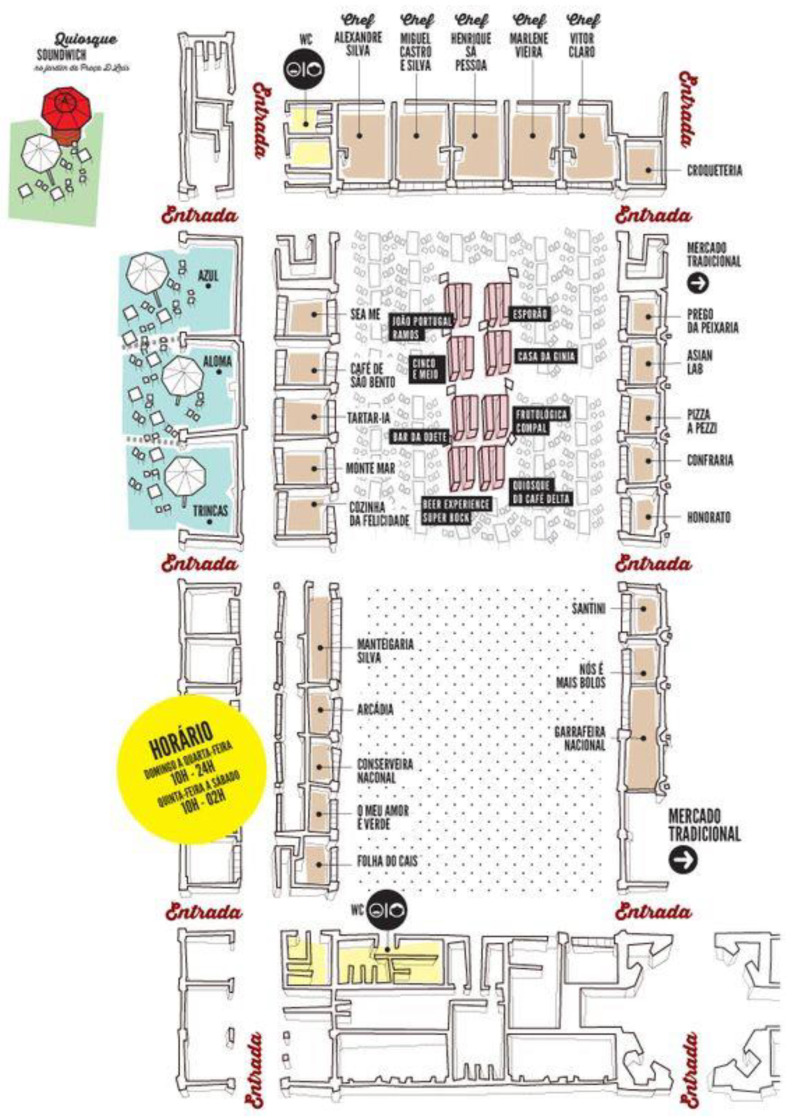
Time Out Market Lisbon food court.

Currently, Time Out Market Lisbon has 26 restaurants, eight bars, a dozen stores, and a high-end music venue, which gathers some of the best known (and oldest) sellers of meat, fish, fruit, and flowers in the city (Figure 2). In the words of Ana Alcobia, market director, who was interviewed prior to this study, a balance was achieved between traditional vendors and restoration concepts, in addition to maintaining the building’s original architecture, with its immediate surroundings and the entire neighborhood of Cais do Sodré.

This reconciliation of the Time Out Market Lisbon with the city, projecting the traditional market among residents and giving it back its main function, which is supply, is now leveraged by other restoration projects. At the same time, the success of the project catapulted the market as a city tourist attraction, not only in terms of gastronomy, but also in terms of heritage and culture.

## 4. Materials and Methods

In this section, we discuss the methodological issues, including the sample description and the instruments used to measure the perception of respondents about the location, food quality, food neophilia, market involvement, place as a cultural attraction, satisfaction, and the intention to revisit the locale. It will also present the data collection procedures. For the study, a quantitative research approach was chosen. It makes use of statistical methods to link food market attributes as a factor in visitors’ satisfaction and revisit intention of gastronomic food markets.

### 4.1. Participants

The research was conducted using a random sampling of 357 visitors of Time Out Market Lisbon, most of whom were male (51.3%). The ages of the participants ranged from 18 to 77 years old (M = 31.76; SD = 11.46) and more than half have academic qualifications at the degree level or higher (69.5%). It was also possible to verify that only 9.5% of the visitors have Portuguese nationality. It was also found that 69.5% of visitors stayed in Lisbon between three and seven days (M = 6.16; SD = 4.35) and that 43.4% (M = 3.71; SD = 3.19) came in groups consisting of three to five people (Table 1).

### 4.2. Measures

The variables of the proposed research model were measured through 27 items by adapting the instruments compilated by Dimitrovski and Crespí-Vallbona [17] based on the items developed by Kim and Eves [46] (e.g., Experiencing the Time Out Lisbon market gives me the opportunity to increase my knowledge about different cultures), Kim et al. [36] (e.g., I like foods from different cultures), Mason and Paggiaro [47] (e.g., I intend to revisit Time Out Lisbon market again), Organ et al. [48] (e.g., I like discussing food products with local producers) and Wan and Chan [49] (e.g., Time Out Lisbon market is in the heart of the city). The respondents expressed their level of agreement with the items using a five-point Likert scale (1—strongly disagree; 5—strongly agree).

### 4.3. Procedures

The researchers made contact with market visitors during their visit and the questionnaires, which took about seven minutes, were administered at the exit door of the food market after the experience. The data were collected between the 5th and the 12th of December of 2022. Subsequently, the data were analyzed using the statistical software SPSS (Statistical Package for the Social Sciences, version 28) and AMOS (Analysis of Moment Structures, version 28).

## 5. Results

The data analysis began with the study of the reliability of the instruments used, having verified that they present an adequate internal consistency with Cronbach’s Alpha coefficients oscillating between 0.79 and 0.90 (Table 2).

To determine the characteristic of the Time Out Market Lisbon that had the greatest impact on visitors’ satisfaction and to validate the first study hypothesis, a multiple linear regression was performed. Observing Table 3, it is possible to verify that the model was linear and statistically significant [*F*_(4,352)_ = 434.026, *p* < 0.001] and that 83.0% of the visitors’ satisfaction was explained by the location, food quality, food neophilia, and market involvement. However, it was found that food neophilia was the variable that had the greatest impact on the visitors’ satisfaction with Time Out Market Lisbon, explaining 12.81% of its variation.

Next, an attempt was made to determine if the intention to revisit was influenced by the visitors’ satisfaction level with Time Out Market Lisbon and it was found that 70.4% of the desire to return was explained by the visitors’ satisfaction with the gastronomic food market. In addition to confirming the linearity of the model [*F*_(1,355)_ = 848.150, *p* < 0.001], the results revealed that the greater the satisfaction of the visitors, the greater their desire to return (β = 0.840, t = −29.123, *p* < 0.001), which validated the second research hypothesis.

We also analyzed whether the model outlined fits the sample under study. For this purpose, a path analysis was carried out, which allowed the description of all the existing relationships between the constructs involved in the analysis. The moderation model of the visitors’ satisfaction, the place as a cultural attraction, and the influence of these variables on Time Out Market Lisbon revisit intention was evaluated using a structural equation model with a moderating effect. The latent moderation factor was defined by the product, in pairs of the items that constitute the visitors’ satisfaction and place as cultural attraction factors. 

The adjustment of the model was developed in two stages: the first to validate the measurement model and the second to adjust the moderation model. In assessing the quality of the adjustment, the CFI and GFI indices were used, considering that these values indicated a good adjustment for values greater than 0.90. A confidence interval (CI) for the RMSEA at 90% with an upper limit of less than 0.10 was considered to indicate a reasonable fit and that the fit is good when the RMSEA is less than 0.08. The significance of the moderation effect was evaluated with a test for the significance of the trajectory coefficient associated with the moderation effect [χ^2^_(83)_ = 2.095, *p* < 0.001, CFI = 0.979, GFI = 0.939, RMSEA = 0.055, LO90 = 0.037, HI90 = 0.061].

**Figure 3:**
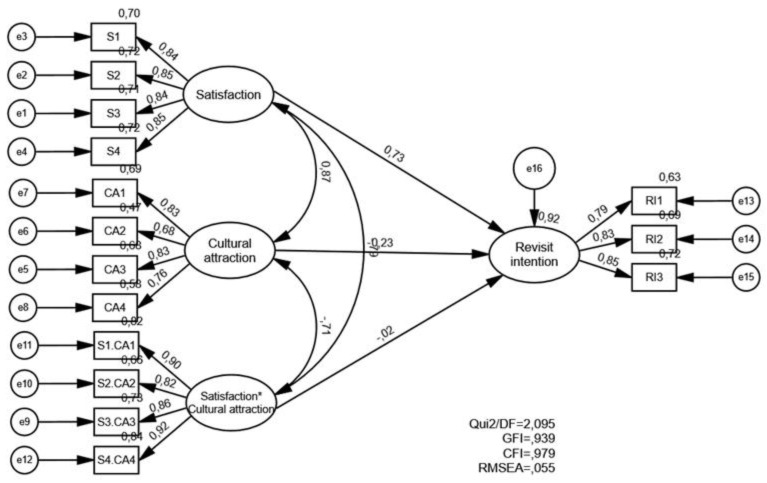
Moderating role of viewing the market as a cultural attraction in the relationship between visitors’ satisfaction and Lisbon Time Out revisit intention.

Figure 3 illustrates the standardized estimates of the parameters from both the measurement model (factor weights) and the structural model of moderation. A moderating effect of visitors’ satisfaction on the market as a cultural attraction was observed in the Time Out Market Lisbon revisit intention (β = −0.020, *p* < 0.001). As the moderation effect was negative, it can be stated that the higher the level of visitors’ satisfaction, the lower the effect of the perception of place as a cultural attraction on the Time Out Market Lisbon revisit intention. There were also statistically significant direct effects of visitors’ satisfaction and viewing the place as a cultural attraction.

To assess the measurement quality of the instruments, the composite reliability (CR) and average variance extracted (AVE) were calculated and the values were shown to be adequate. Observing Table 4, it is possible to verify that the CR values of all the constructs were equal to or greater than 0.75, thus revealing good reliability. The AVE values were greater than 0.50 supporting convergent validity. Discriminant validity was also ensured from the results, since the ASV and MSV presented values below the AVE [50].

The results showed that food neophilia is the characteristic of Time Out Market Lisbon that had the greatest impact on visitors’ satisfaction, which in turn positively influenced the visitors’ intention to revisit. It was also found that the visitors’ perception of the place as a cultural attraction moderated the relationship between visitors’ satisfaction and Time Out Market Lisbon revisit intention.

## 6. Discussion

Despite witnessing a revitalization of traditional urban food markets all over the world, through transformation into tourist attractions, little attention has been paid to the investigation of the factors that shape visitors’ satisfaction. Hence, satisfaction must be analyzed in all aspects that make up the visit to food markets and not just in terms of the quality of the food. Recently, one of the issues that has aroused the greatest interest among researchers is the impact of food neophilia in tourism activity [35,40,51]. The cultural aptitude of the food markets and its impact on the overall satisfaction of the visit has also been the subject of several studies [17,24,52]. That is, although visitors are attracted by the local culinary offering that exist in urban food markets, the markets are also viewed by visitors as a genuine representation of local culture. 

The present study is based on the work of Dimitrovski and Crespí-Vallbona [17] in the La Boqueria food market, in Barcelona; we adapted their research model in order to understand what are the factors that determine the success of a food/gastronomic market. The study focused on Time Out Market Lisbon, considered a success story, which has been replicated in other cities around the world. In addition to corroborating and expanding the variables presented by Dimitrovski and Crespí-Vallbona [17], the originality of the study lies in correlating visitor satisfaction with the market as a cultural attraction and the intention to revisit. 

The results of our study suggest that the experience in food markets is multidimensional, where location, market involvement, food quality, and food neophilia are factors that contribute positively to the experience of visiting the market. These findings corroborate the study by Dimitrovski and Crespí-Vallbona [17,24] on the La Boqueria market in Barcelona. Nevertheless, food neophilia was the dimension that contributed the most to visitor satisfaction. This result is in line with the findings of Baah et al. [35] that highlight the critical role of neophilia regarding international tourists’ attitudes toward indigenous cuisine. It can therefore be concluded that, although visitors identify the location and involvement of the market, the hedonic sensory adventure of discovering new flavors commands the visit to Time Out Market Lisbon. Thus, there seems to be agreement between various studies of the hedonic vocation of urban food markets, as a catalyst for the visit and its contribution for the intention to revisit [15,17,30].

In view of these results, it was considered relevant to determine the moderating effect of the cultural attraction on the relationship between visitor satisfaction and the intention to revisit Time Out Market Lisbon and it was found that the more satisfied visitors felt, the smaller the effect of the perception of the place as a cultural attraction on the intention of revisiting Time Out Lisbon. These conclusions are in line with the studies developed by Chi, Huang, and Nguyen [53] according to which the greater the satisfaction of visitors with the existing local gastronomic offerings in urban food markets, the greater their desire to return to the same destination. Previous research showed that overall satisfaction had a significantly positive effect on the intention to revisit and become a repeat visitor [54]. Liu [55] went further and stated that although local culture has a significant impact on visitors’ intentions to return to the destination, it is the memorable experiences that motivate tourists to return. In the same line, Viet, Dang, and Nguyen [56] argued that despite the importance attributed to the cultural aspects of the place, satisfaction with the experience is the factor that most influences the intention to search the place and recommend the destination to other people, as verified in the present study.

## 7. Conclusions

The importance attributed to gastronomic tourism has grown significantly in recent years. From the moment that food began to gain a connotation different from that of the simple act of satisfying a human need and began to offer pleasures, memories, and sensations associated with the social, cultural, and historical context of certain peoples and/or regions, it motivated the area of tourism to study and understand its particularities [57]. Thus, gastronomic tourism can be identified as a powerful tool that encourages tourist flows and contributes to the development of localities. This attraction can be operationalized in several ways (e.g., regional cuisine events, itineraries, routes, and gastronomic circuits) but a constant offer of tourist food services must always be maintained. What is offered is much more than a delicacy, it is a gustatory and cultural experience, which brings food consumption closer to the conception of symbolic consumption [55].

The present study is part of this theme and sought to analyze the perceptions of tourists about gastronomic food markets in an urban context, specifically the case of Time Out Market Lisbon. For this purpose, we sought the opinion of the participants about the location, the quality of the food, food neophilia, involvement with the market, and the place as a cultural attraction. It was also intended to determine how these variables contribute to visitor satisfaction and their intention to revisit the site. 

The results showed that food neophilia was the attribute of Time Out Market Lisbon that most influenced visitor satisfaction. Satisfaction is a necessary condition to return to the place and to transmit this information to relatives and/or colleagues [58]. Visitors to these types of events recognize the value of gastronomy as a means of socializing and a means of sharing life with others [59]. It was also possible to verify that the visitors’ perception of the place as a cultural attraction moderated the relationship between visitor satisfaction and the intention to revisit Time Out Market Lisbon. These results can be explained by the fact that gastronomy has been incorporated as part of the cultural identity of the country/region [60]. One of the main components of a gastronomic brand are gastronomic events (e.g., markets) that allow visitors to not only enjoy the food during their visit, but also to carry memories and the desire to cook in similar ways, while planning to visit other restaurants in a city [61].

### 7.1. Theoretical and Practical Implications

The results of our study show the need to consider urban food markets, now also transformed into gastronomic markets, as references for the promotion of indigenous ingredients and cuisine, as well as local culture. This cultural vocation of the food markets reinforces the desire of the management of Time Out Market Lisbon, which in an interview for our research confided to us that it has plans to increasingly include cultural activities in its programming. In a broader perspective, the local authorities responsible for tourism promotion should see urban food markets as a vehicle for the revitalization of cities, where, in line with the desire that visitors express in experiencing new ingredients and flavors, other tourism modalities emerge, such as history and local culture. Bearing in mind that Time Out Market Lisbon has served as an inspiration for similar concepts in various locations around the globe, taking advantage of the cultural vocation of spaces can also be followed by the brand, to promote local culture and thus enrich the visit experience. Furthermore, the findings of this study can provide a motto not only for restaurant businesses, but also for the food industry, to better understand the impact of food neophilia on overall customer and consumer satisfaction.

### 7.2. Limitations and Future Research

Despite the success of the concept developed at Time Out Market Lisbon, which continues to be the reference for opening similar spaces in cities with great tourist potential, the results of this study cannot be generalized because the perception of the experience on similar food markets may differ according to the culture and social background, and different generations. Hence, it would also be interesting to apply the same methodology to food markets in other locations, cities, and countries.

Therefore, it would be equally worthwhile to apply this same methodology not only to other food markets in other place, cities, and countries, but also to local restaurants which use native products and recipes, and which are being visited by tourists from other countries and cultures.

Regarding Time Out Market Lisbon, it would be interesting to apply cluster analysis, in order to better understand the possible different profiles of visitors and behavioral patterns. Additionally, future research could include other constructs like social interaction and hospitality. In future research, a qualitative methodology could also be adopted, to complement the quantitative data, namely, to understand the inhabitants’ opinion about the importance of the market on the neighborhood’s sustainability, as well as the traditional vendors’ and restaurant owners’ statements regarding the commercial exploration of neophilia impact.

## Figures and Tables

**Table 1:** Sample sociodemographic characteristics (n = 357).

Sociodemographic Characteristics	Number of Respondents (%)
Gender Male Female	183 (51.3) 174 (48.7)
Age (years) 18–24 25–34 35–44 45–54 >55	95 (26.6) 162 (45.4) 48 (13.4) 29 (8.2) 23 (6.4)
Education High school graduate Bachelor’s degree Master’s degree Ph.D. degree	109 (30.5) 157 (44.0) 84 (23.5) 7 (2.0)
Nationality German American Brazilian Canadian Chinese Spanish French Dutch English Irish Italian Portuguese	42 (11.8) 26 (7.3) 15 (4.2) 14 (3.9) 20 (5.6) 27 (7.6) 43 (12.0) 15 (4.2) 82 (23.0) 15 (4.2) 24 (6.7) 34 (9.5)
Length of stay in Lisbon Weekend (1–2 days) One week (3–7 days) Two weeks (8–15 days) 16 days or more	36 (10.1) 248 (69.5) 55 (15.4) 18 (5.0)
Visitors (including the respondent) Themselves 2 people 3–5 people ≥6 people	59 (16.5) 91 (25.5) 155 (43.4) 52 (14.6)

**Table 2:** Reliability analysis.

Variables	Cronbach’s Alpha
Location Food quality Food neophilia Market involvement Place as cultural attraction Satisfaction of visitors Lisbon Time Out revisit intention	0.79 0.87 0.88 0.87 0.86 0.90 0.85

**Table 3:** Explanatory variables of visitors’ satisfaction.

Explanatory Variables	Satisfaction of Visitors (β)	R^2^ Semi-Partial (%)
Location Food quality Food neophilia Market involvement	0.116 * 0.237 ** 0.357 ** 0.259 **	2.40 6.05 12.81 7.12
Adjusted R Square	0.830	
*F*_(4,352)_	434.026 **	

Note: * *p* < 0.05; ** *p* < 0.001.

**Table 4:** Composite reliability, and convergent and discriminant validity.

Variables	CR	AVE	MSV	ASV
Satisfaction of visitors Place as cultural attraction Time Out Market Lisbon revisit intention	0.93 0.90 0.91	0.88 0.81 0.88	0.75 0.79 0.81	0.82 0.77 0.84

Note: CR = composite reliability; AVE = average variance extracted; MSV = maximum shared variance; ASV = average shared variance.

## Data Availability

Data is unavailable due to privacy restrictions.
